# Supplementary material for: Chromothripsis during telomere crisis is independent of NHEJ, and consistent with a replicative origin
Source: Genome Res. 2019 May;29(5):737–49. doi: 10.1101/gr.240705.118 (PMC6499312; doi:10.1101/gr.240705.118)
Supplement: Supplemental Material [file supp_gr.240705.118_Supplemental_file_1.zip › contigs/annotated_contigs/DB79/contig.2.DB79_length_572_mean_cov_14.1993006993.docx]

**DB79_length_572_mean_cov_14.1993006993**

```
AAGGTGGTGTTTTCGTGGCGGTC|GTAAAAAAAAACCTAGTGTTATTGAAGCCACTGAGTTTCAGGATCATTGTTAGTGCAGCATAACC
                        >chr9:98456676-98456916 + E=1e-129
TAGACTTACCCTGACTGATACAGTTATTCTTTAAAAATGAACCTTTCAAAAACACCCATCTTCTCTAAGCTTTTATGGTATTTATGAAA

ACATCTCACACTCCCAATGCAGCCTATGTTTTCTTAACTAGTGTTCTAGGCAGTTCAGTTGTTATTTGTTTGTTTATAATGAGCTT|GC

CATTCAGGAGGGGTTTTTAGGTGCTCTGCA|TCCTGATCTCGTGATCTGCCTGTCTTGGCCTCCCAAAGTGCTGGGATTACAGGTGTGA
                               >chr9:131880556-131880833 + E=7e-119
GCCACCACGCCCAGCGTATTTGGGTTGTTTCTAATTAATAAAAAATTTTTTTATTTGAGACAGAGTTTCGCTCTTCTTGCCCAAGCTGG

AGTGCAATGGTGTGATCTTGGCTCACTGCAACCTCTGCCTCCTGGTTCAAGCAATTCTCCTGCCTCAGCCTCCCAAGTAGCTGGGATTA

CAGGCATGCGCCACCACACCTGAATAATTTTATATTTTTAG
```
